# Measurement Accuracy and Improvement of Thematic Information from Unmanned Aerial System Sensor Products in Cultural Heritage Applications

**DOI:** 10.3390/jimaging10020034

**Published:** 2024-01-28

**Authors:** Dimitris Kaimaris

**Affiliations:** School of Spatial Planning and Development, Aristotle University of Thessaloniki, 54124 Thessaloniki, Greece; kaimaris@auth.gr

**Keywords:** Unmanned Aerial System, digital surface model, orthophotomosaic, accuracy, image fusion, correlation table, ERGAS index, spectral deviation, image quality

## Abstract

In the context of producing a digital surface model (DSM) and an orthophotomosaic of a study area, a modern Unmanned Aerial System (UAS) allows us to reduce the time required both for primary data collection in the field and for data processing in the office. It features sophisticated sensors and systems, is easy to use and its products come with excellent horizontal and vertical accuracy. In this study, the UAS WingtraOne GEN II with RGB sensor (42 Mpixel), multispectral (MS) sensor (1.2 Mpixel) and built-in multi-frequency PPK GNSS antenna (for the high accuracy calculation of the coordinates of the centers of the received images) is used. The first objective is to test and compare the accuracy of the DSMs and orthophotomosaics generated from the UAS RGB sensor images when image processing is performed using only the PPK system measurements (without Ground Control Points (GCPs)), or when processing is performed using only GCPs. For this purpose, 20 GCPs and 20 Check Points (CPs) were measured in the field. The results show that the horizontal accuracy of orthophotomosaics is similar in both processing cases. The vertical accuracy is better in the case of image processing using only the GCPs, but that is subject to change, as the survey was only conducted at one location. The second objective is to perform image fusion using the images of the above two UAS sensors and to control the spectral information transferred from the MS to the fused images. The study was carried out at three archaeological sites (Northern Greece). The combined study of the correlation matrix and the ERGAS index value at each location reveals that the process of improving the spatial resolution of MS orthophotomosaics leads to suitable fused images for classification, and therefore image fusion can be performed by utilizing the images from the two sensors.

## 1. Introduction

A typical process for collecting and processing photogrammetric data includes specific steps. In brief, Ground Control Points (GCPs) and Check Points (CPs) [1,2,3] are first selected in the field, followed by the determination of their coordinates (x, y and z) by means of a surveying instrument (e.g., Global Navigation Satellite System (GNSS)). GCPs are required to resolve the triangulation, while CPs are required to monitor the products produced (Digital Surface Model (DSM) and orthophotomosaic) [4]. Next, the required images are collected and then processed via an appropriate photogrammetric or remote sensing software, allowing at last the production of the DSM and the orthophotomosaic of the study area [4,5].

The products, the DSM and orthophotomosaic, should be tested using CPs to determine their actual horizontal and vertical accuracy. Coordinates x’, y’ and z’ of the CPs are digitally collected (from, e.g., a geographic information system or photogrammetric or remote sensing software) from these products in order to be compared with the coordinates (x, y, z) of the same CPs measured in the field (e.g., via GNSS).

The principal methods of product evaluation are the mean, the standard deviation and the Root Mean Square Error [4,5,6,7]. Furthermore, when we are dealing with normally distributed data, then the analysis of variance (ANOVA) performs hypothesis tests to determine the differences in the mean values and standard deviations of the various data sets (x-measurement on the product and x-measurement in the field, y-measurement on the product and y-measurement in the field, z-measurement on the product and z-measurement in the field) [8].

Currently, aerial surveys are mainly carried out with the use of an Unmanned Aerial System (UAS). This is due to the fact that these systems offer ease of use, product accuracy and automation in aerial data collection processes. Modern UASs feature sophisticated sensors and systems that minimize working time [9] both in the field and in the office. The working time in the field is significantly reduced as, according to the UAS manufacturers, either the collection of GCPs and CPs is not necessary, or the number is very small when the UAS is equipped, e.g., with a multi-frequency Post-Processing Kinematic (PPK) GNSS receiver [10]. In the office, the elimination of manual selection of GCPs or automatic finding of GCPs in images that then need to be checked to confirm that they were correctly marked reduces processing time [8]. However, in PPK or Real Time Kinematic (RTK) systems, inherent high systematic errors occur in the calculation of the Z coordinates [5].

In several projects where a UAS is equipped with RTK or PPK, it has been observed that processing without the use of GCPs leads to good horizontal accuracy (comparable to the accuracy achieved with the exclusive use of GCPs), but considerably lower altimetric accuracy compared to that achieved by the exclusive use of GCPs. In these applications, there are a variety of different terrains in the areas to be mapped (smooth to rugged terrain), a structured to unstructured mapping surface, different flight heights (from 30 m to 120 m), different sensors, classic image collection strips perpendicular to each other in the same flight, different UASs (multi-rotor, fixed-wing), etc. [4,11,12,13,14,15,16,17,18]. However, there are also studies (though fewer) that report that the use of RTK or PPK (processing without GCPs) results in products of equal or better accuracy on all three axes as opposed to processing with the exclusive use of GCPs [19,20,21,22].

In the present study, the UAS WingtraOne GEN II with RGB sensor (42 Mpixel) and built-in multi-frequency PPK GNSS antenna was used to calculate with a high level of accuracy the coordinates of the centers of the images received [10].

The first objective of this study is to test the accuracy of the DSM and the orthophotomosaic of the UAS RGB sensor by exploiting a large number of CPs when a solution is applied:

- Without GCPs (direct georeferencing), but with known X, Y and Z coordinates of the image centers (PPK utilization);
- Using only GCPs (no known X, Y or Z values of the image centers).

**Figure 1:**
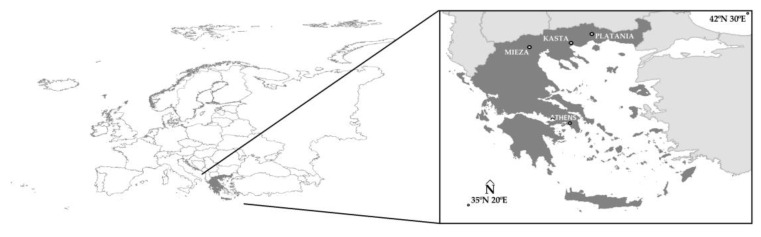
The location of Greece in Europe and the locations of the archaeological sites of the Acropolis of Platanias, the Ancient Theater of Mieza and the Kasta Mound.

The above shows whether classical processing with GCPs leads to better results compared to processing without the use of GCPs (using only PPK data) or vice versa. To enable this test, 20 GCPs and 20 CPs were measured in the field by means of GNSS (Topcon HiPer SR, Tokyo, Japan). For each of the above two processing cases, the coordinates (x’, y’, z’) of the CPs were extracted from the products (DSM and orthoimagery) and then compared with the measurements (using GNSS) of their coordinates (x, y, z) in the field. This research was carried out at the archaeological site of the Acropolis of Platanias (North Greece, Figure 1).

From the very early years of the emergence of remote sensing science, one of the key processes for processing satellite images was image fusion. Methodological image fusion procedures allow us to improve the spatial resolution of the multispectral (MS) image by utilizing the panchromatic (PAN) image with better spatial resolution, while trying to preserve to a large extent the spectral information of the original MS image transferred to the fused image [23,24,25,26,27,28,29,30,31,32,33,34,35,36,37,38,39,40,41].

In the case of UASs, MS sensors do not feature a PAN sensor. The only exception is the MicaSense RedEdge-P (with a high-resolution PAN band), which made its appearance a few months ago.

The UAS used in this study can also make use of an MS sensor (1.2 Mpixel, Bands: RGB, Blue, Green, Red, RedEdge, Near Infrared (NIR)), replacing the RGB sensor (42 Mpixel) and performing a new flight to capture the study area.

In previous papers, image fusion was performed using RGB and MS images from the same sensor (Sequoia+ by Parrot) or different sensors (RGB images from Phantom 4 and MS images from Sequoia+ by Parrot) for the UAS. These efforts have demonstrated that it is feasible to improve the spatial resolution of MS images, while preserving a reasonable amount of the spectral information of the original MS images transferred to the fused images [8,42]. The minimum allowable flight height with the MS sensor (1.2 Mpixel) on the WingtraOne GEN II UAS is 100 m. This results in a spatial resolution of the MS images of about 7 cm. The spatial resolution of the RGB sensor (42 Mpixel) for the minimum allowable flight height of 60 m (the minimum allowable flight heights for the two sensors are different) is about 1 cm. Thus, it is interesting to produce fused images with a spatial resolution of about 1 cm, because in many archaeological investigations this spatial resolution is required.

The second objective of this paper is to perform image fusion using the images of the two sensors (RGB 42 Mpixel and MS 1.2 Mpixel) of the UAS, and to control the spectral information of the original MS images transferred to the fused images. The research took place in three archaeological sites, the Acropolis of Platanias, the Ancient Theater of Mieza and the Kasta Mound (the locations are in Northern Greece, Figure 1).

## 2. Areas of Study

The Acropolis of Platanias (41°11′05.4″ N 24°26′03.2″ E) is located in the prefecture of Drama (Northern Greece, Figure 1). Archaeological research has revealed the existence and use of the site of the acropolis since prehistoric times until late Roman antiquity. It is an acropolis of an ellipsoidal shape at an altitude of about 650 m to 670 m with a perimeter of about 270 m, built on a natural rock. The height of the walls varies from 2.3 m to 2.5 m. The first phase of the acropolis dates back to prehistoric times, while in its second phase it was used by cattle breeders. In its third phase, it was developed by the Greek king of Macedonia, Philip II (382 BC–336 BC), as a point of control for the wider region. The fourth phase of the acropolis dates back to Roman times; the fifth phase is linked to the construction of the dormitories and storage areas of the 3rd century AD and the sixth phase is linked to coins and other findings of the 6th century AD, which testify to the presence of a small garrison in the acropolis [43].

The Ancient Theater of Mieza (Northern Greece, Figure 1) belongs to the ancient city of Votiaia Mieza (40°38′38.6″ N 22°07′21.3″ E). It was discovered in 1992 during the excavation of an underground irrigation network. It is located on the slope of a low hill, facing east. Most of its hollow has been carved in the natural soft limestone, on which the rows of seats have been placed. Most of the stones of the first seven rows have been preserved. Carvings in the natural rock, however, confirm the existence of at least 19 levels. Four staircases divide it into five stands. The orchestra is semi-circular in shape with a diameter of about 22 m. The stage consists of the main stage building and the proscenium. The southern backstage and small parts of the walls in the southern part of the stage are preserved and found at the level of its foundation. The earliest phase of the monument dates back to the Hellenistic period. Following the middle of the 2nd century BC, a new late Hellenistic-early Roman theater was built. The partial collapse of the hollow and part of the stage, probably in the 2nd century AD, led to makeshift repairs. According to coins and pottery, the theatre must have been in operation up to the 4th century AD [44].

Inside the Kasta Mound (Amphipolis, Northern Greece, Figure 1), a Macedonian burial monument was discovered dating to the last quarter of the 4th century BC (40°50′21.5″ N 23°51′44.9″ E). In the mid-1950s and until the 1970s, excavations were carried out in the upper part of the mound, bringing to light a set of modest tombs dating back to the Iron Age. Excavation of the perimeter of the site began again in 2012, and in 2014 the first findings were unearthed on the south side of the mound, i.e., the entrance to the burial monument. Three chambers were then discovered (a total of four rooms including the entrance and the stairs to the interior of the tomb). The marble enclosure of the circular mound has a perimeter of 497 m, a height of 3 m and an area of about 20,000 sq.m., and it was constructed using approximately 2500 m^3^ of Thassos marble. In its entirety, it is the largest burial monument discovered in Greece, and one of the most important international archaeological discoveries of 2014. In short, at the entrance of the burial monument, there is a door above which stand two marble sphinxes. Inside the mound (first chamber) there are two “Caryatids” resting on piers. In the second chamber there is a floor mosaic depicting “The Abduction of Persephone by Pluto”. In the third chamber, a tomb was found with bones belonging to five persons (the skeletons are not whole) and the remains of a horse skeleton. According to the excavation team, the monument was constructed by Deinocrates (Greek architect and technical advisor of Alexander the Great, known for many works, such as the urban planning and construction of the city of Alexandria, the funeral pyre of Hephaestion and the reconstruction of the Temple of Artemis at Ephesus) and commissioned by Alexander the Great [45].

## 3. Equipment

**Figure 2:**
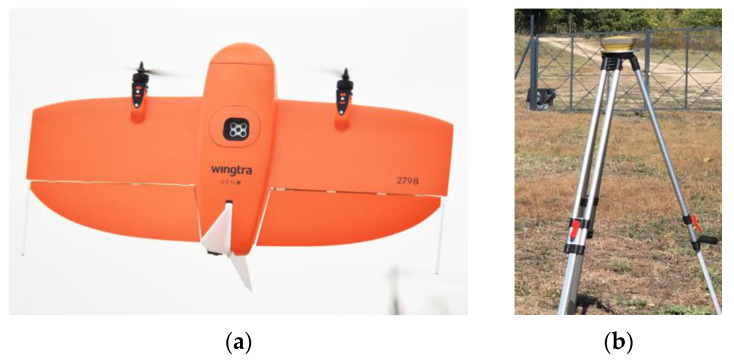
(**a**) The UAS WingtraOne GEN II; (**b**) the GNSS Topcon HiPer SR mounted on a tripod.

For the collection of the aerial images from the three archaeological sites, the UAS WingtraOne GEN II of Wingtra was used, while for the measurement of the GCPs and CPs at the Acropolis of Platanias, the GNSS Topcon HiPer SR was used (horizontal and vertical accuracy of real-time positioning of approximately 10 mm and 15 mm, respectively; GPS: L1, L2, L2C, GLONASS: L1, L2, 2C, SBAS-QZAA: L1, L2C) (Figure 2). No ground targets were used, as there were plenty of distinctive points on the weathered stones (the building material of the acropolis) that were distinct and easily identifiable.

The WingtraOne GEN II is a fixed-wing vertical takeoff and landing (VTOL) UAS, weighing 3.7 kg and measuring 125 × 68 × 12 cm. The maximum flight time is 59 min. For the calculation of the coordinates of the centers of the images received, it utilizes a built-in multi-frequency PPK GNSS antenna (GPS: L1, L2; GLONASS: L1, L2; Galileo: L1; BeiDou: L1). The flight plan and parameters are defined through the WingtraPilot^©^ 2.11 software. It is equipped with one RGB and one MS sensor (Table 1).

## 4. Materials

### 4.1. Flight Plans and Image Collection

**Figure 3:**
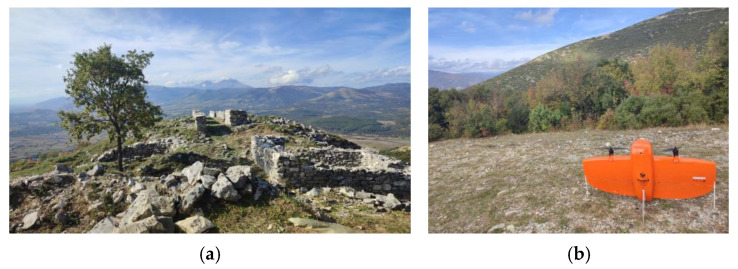
(**a**) The Acropolis of Platanias; (**b**) the UAS.

**Figure 4:**
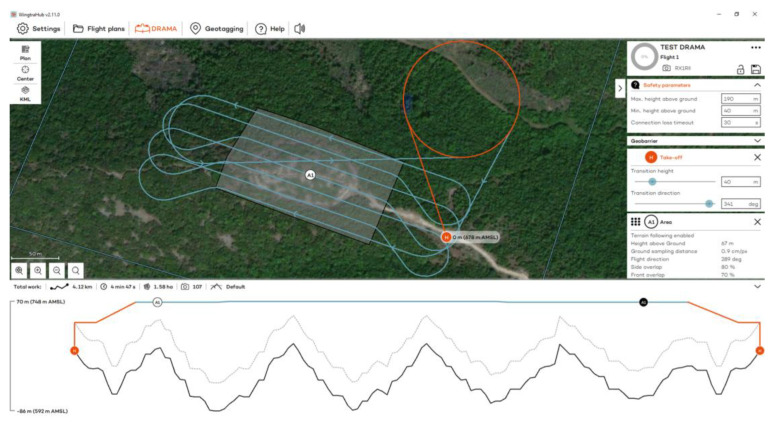
The flight plan at the Acropolis of Platanias for the RGB sensor.

**Figure 5:**
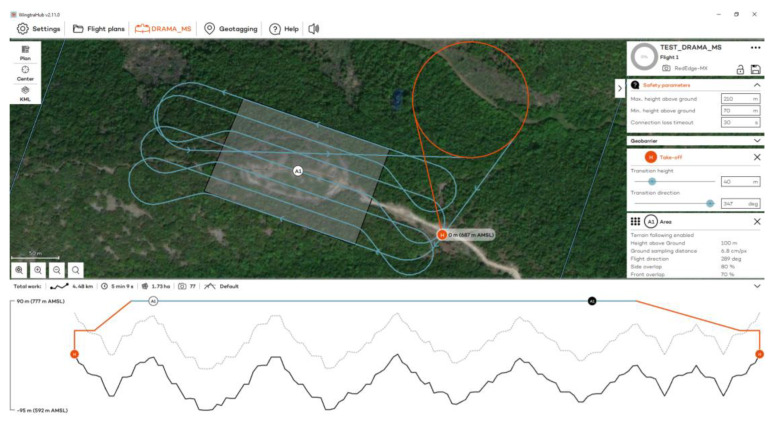
The flight plan at the Acropolis of Platanias for the MS sensor.

Flights to the Acropolis of Platanias took place on 3 November 2023 at 12:30 p.m., using RGB and MS sensors (Figure 3). Flights were designed with 80% side and 70% front image overlap (Figure 4 and Figure 5). Seven strips were developed for the RGB and MS sensors. Flight height was 67 m in the case of the RGB and 100 m in the case of the MS sensor (the minimum allowed flight height for the RGB sensor is 60 m and for the MS sensor is 100 m). The expected spatial resolution of the RBG images was 0.9 cm, and of the MS images 6.8 cm. Flight time was 4 min and 47 s with the RGB sensor and 5 min and 9 s with the MS sensor. The images that were collected reached 107 RGB and 77 MS images.

**Figure 6:**
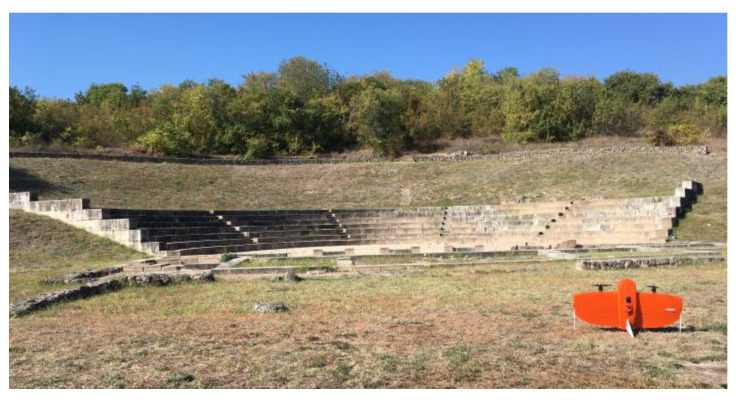
The Ancient Theater of Mieza and the UAS.

**Figure 7:**
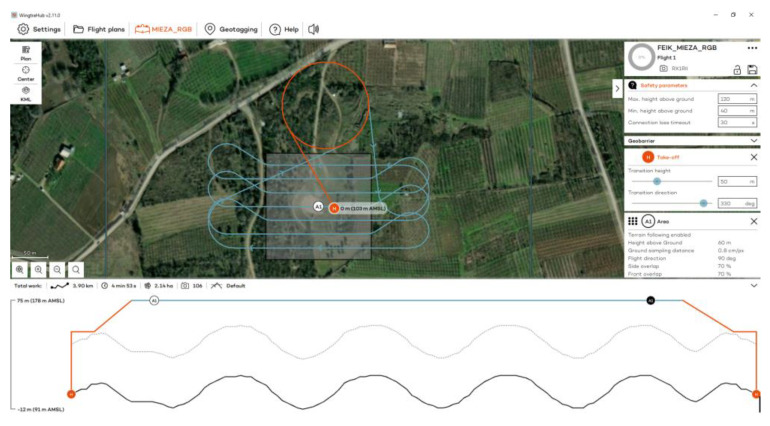
The flight plan at the Ancient Theater of Mieza for the RGB sensor.

**Figure 8:**
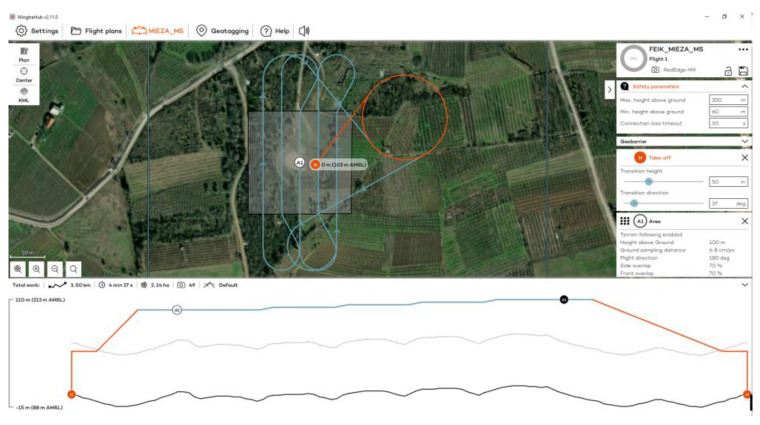
The flight plan at the Ancient Theater of Mieza for the MS sensor.

The flights at the Ancient Theater of Mieza took place on 13 October 2023 at 11:00 a.m., using RGB and MS sensors (Figure 6). Flights were designed with 70% side and front image overlap (Figure 7 and Figure 8). Seven strips were developed for the RGB and five strips for the MS sensor. Flight height was 60 m in the case of the RGB and 100 m in the case of the MS sensor. The expected spatial resolution of the RBG images was 0.8 cm and of the MS images 6.8 cm. Flight time was 4 min and 53 s with the RGB sensor and 4 min and 27 s with the MS sensor. The number of collected images was 106 RGB and 49 MS images.

**Figure 9:**
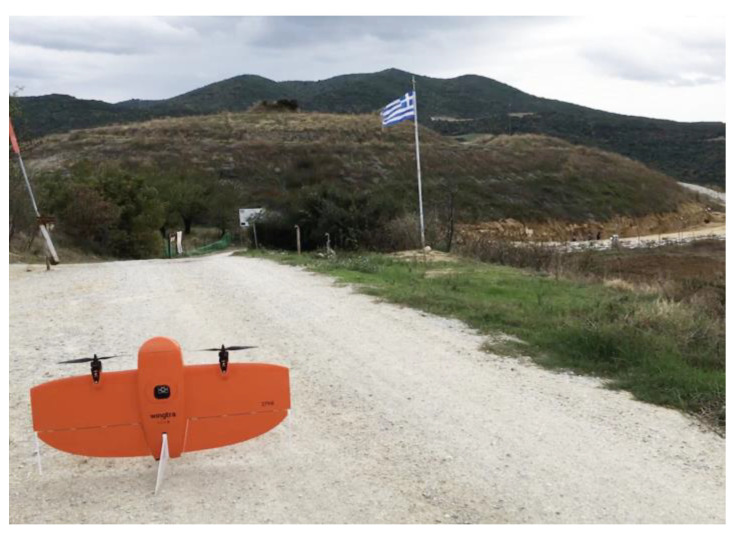
The Kasta Mound and the UAS.

**Figure 10:**
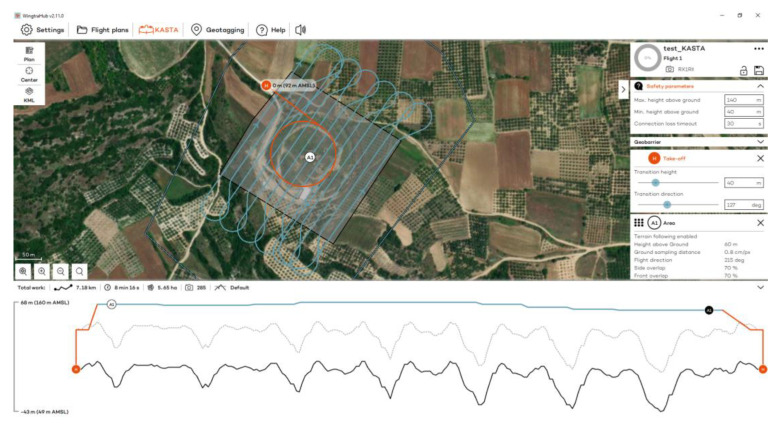
The flight plan at the Kasta Mound for the RGB sensor.

**Figure 11:**
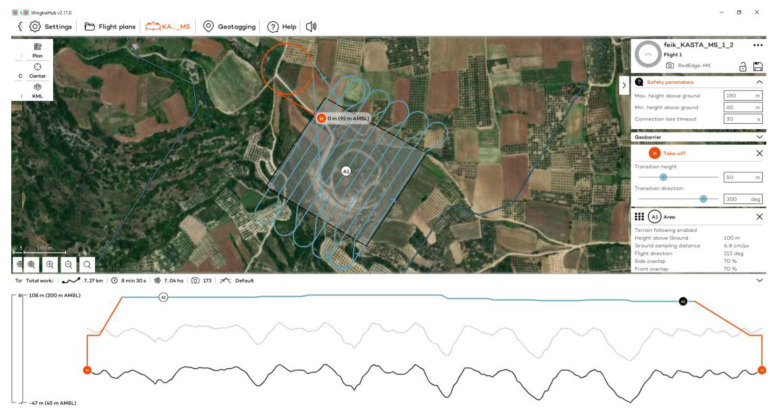
The flight plan at the Kasta Mound for the MS sensor.

The flights at the Kasta Mound took place on 10 November 2023 at 11:30 a.m., using RGB and MS sensors (Figure 9). Flights were designed with 70% side and front image overlap (Figure 10 and Figure 11). A total of 13 strips were developed for the RGB and 11 strips for the MS sensor. Flight height was 60 m in the case of the RGB and 100 m in the case of the MS sensor. The expected spatial resolution of the RBG images was 0.8 cm, and of the MS images 6.8 cm. Flight time was 8 min and 16 s with the RGB sensor and 8 min and 30 s with the MS sensor. The collected images reached 285 RGB and 173 MS images.

### 4.2. Terrestrial Data Collection and Processing

**Figure 12:**
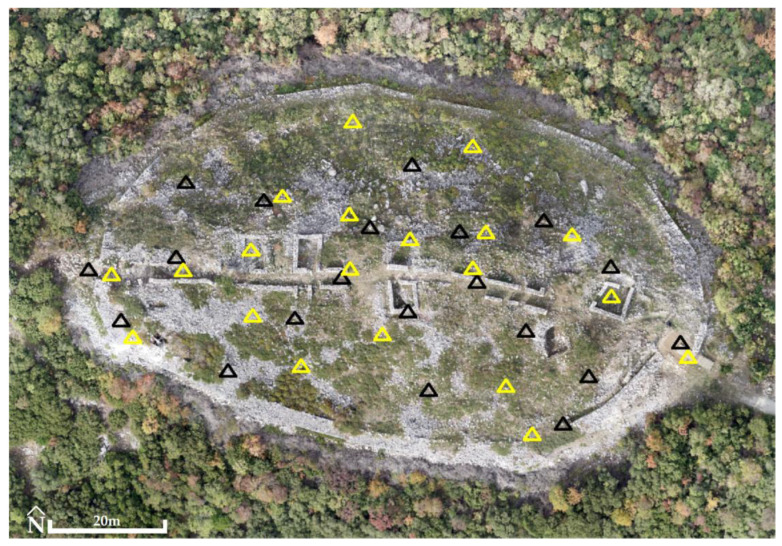
The Acropolis of Platanias (41°11′05.4″ N 24°26′03.2″ E). The distribution of 20 GCPs (triangles in yellow) and 20 CPs (triangles in black) (background: RGB orthophotomosaic, true color).

Prior to the flight at the Acropolis of Platanias, 20 GCPs and 20 CPs (Figure 12, Table 2) were recorded using the GNSS Topcon HiPer SR and the RTK method. Their horizontal and altimetric accuracy in the Greek Geodetic Reference System 87 (GGRS87) were 1.6 cm and 2.4 cm, respectively.

Regarding the GNSS (with Topcon HiPer SR) measurements related to the PPK system of the UAS, initially the x, y and z coordinates of a random point (considered as the base for the subsequent measurements) were measured with millimetric accuracy (1.7 cm horizontal and 2.6 cm vertical) at GGRS87, a short distance from the home position of the UAS, using the RTK method and the network of multiple permanent stations in the country provided by Topcon. Then, using the same GNSS at the same point, continuous position measurements were taken using the Static method for 30 min before the start of the flight, during the flight and for 30 min after the end of the flight. Utilizing the high-precision coordinates of the above point, its Static measurements and the in-flight measurements of the built-in multi-frequency PPK GNSS antenna of the UAS, the coordinates (X, Y and Z) of the reception centers of each image were corrected and calculated at the office (with the same UAS manufacturer’s WintraHub^©^ 2.11 software), finally yielding 3D accuracy in GGRS87 of 2 (horizontal) to 3 cm (vertical).

## 5. Methods and Results

### 5.1. Processing of Images

Processing in Agisoft Metashape Professional^©^ version 2.0.3 consists of fixed steps. First, the images are imported into the software and the GGRS87 coordinate system is defined.

Solely in the case of using the MS sensor, it is necessary immediately after importing the images into the software to calibrate the spectral information using spectral targets. Therefore, before and after the flight, the suitable calibration target of the Micasense RedEdge-MX was imaged. The target was automatically detected using the Agisoft Metashape Professional^©^ and the reflectance values of all spectral bands were calculated [47,48,49,50,51,52,53,54].

Then, when using either the RGB or MS sensor, the alignment of images is performed (align photos with high accuracy) and at the same time, a sparse point cloud model based on matching pixel groups between images is generated. A difference is found at this point, whether using GCPs or not.

When GCPs are used, the process of identifying and marking the GCPs in each image should be initiated. On completion, the Root Mean Square Error for x coordinate (RMSE_x_) (and RMSE_y_, RMSE_z_), the RMSE for x and y coordinates (RMSE_xy_) and the RMSE for x, y and z coordinates (RMSE_xyz_) for all the GCP locations are calculated [55].

When GCPs are not used, after the alignment of images and the production of a sparse point cloud model, the Root Mean Square Error for X coordinate (RMSE_X_) (and RMSE_Y_, RMSE_Z_), the RMSE for X and Y coordinates (RMSE_XY_) and the RMSE for X, Y and Z coordinates (RMSE_XYZ_) for all the sensor locations are calculated [55].

It may be assumed that the above RMSE values provide, roughly, a general idea of the accuracy of the produced DSMs and orthophotomosaics, as these values almost never correspond to the actual accuracy values of the products.

It is worth mentioning here that in parallel with the calculation of RMSE, self-calibration of the sensors could be performed, but was not carried out in any of the processing cases studied. This is because a quality sensor pre-calibration feature is not available, so self-calibration may lead to incorrect calculation of the internal orientation parameters and consequently to large errors in the final products (mainly vertical in DSM and less horizontally in orthophotomosaic) [12,17,56].

Then, when using either the RGB or MS sensor, the dense point cloud is created (build dense cloud; high-quality and aggressive depth filtering). Next, the 3D mesh generation (build mesh) follows, where the point cloud is transformed into an actual 3D surface. The following step is to build the texture (build texture), i.e., the colored overlay of the generated 3D mesh. The last step is to generate a DSM and orthophotomosaic.

**Figure 13:**
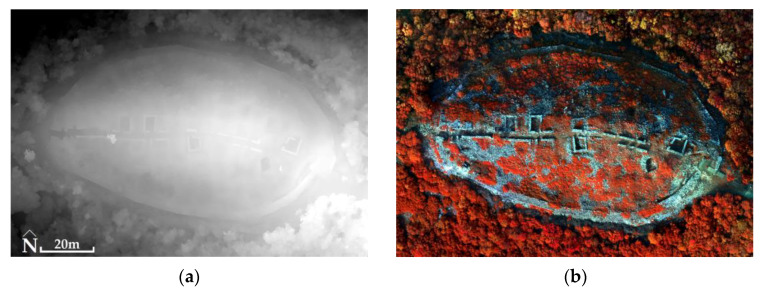
The Acropolis of Platanias (41°11′05.4″ N 24°26′03.2″ E): (**a**) DSM (altitudes: from black color 634 m to white color 674 m) using GCPs in the processing of RGB (for example) images; (**b**) orthophotomosaic (NIR, Green, Blue) without the use of GCPs in the processing of MS (for example) images.

For the Acropolis of Platanias and the RGB images, the RMSE_xyz_ was 2.4 cm in the case of using GCPs, while the RMSE_XYZ_ was 1.2 cm in the case of not using GCPs. The generated products had a spatial resolution of 2.1 cm for DSM (Figure 13) and 1 cm for orthophotomosaic in both processing cases (using or not using GCPs). For MS images, the RMSE_XYZ_ was 1.1 cm (not using GCPs). The generated products had a spatial resolution of 16.7 cm for DSM and 8 cm for orthophotomosaic (Figure 13, Table 3).

**Figure 14:**
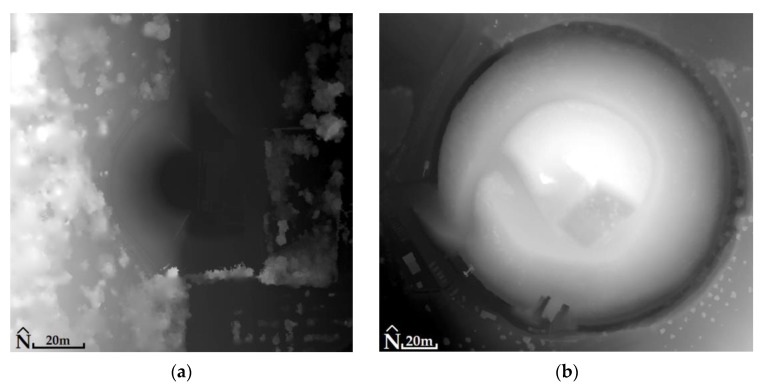
(**a**) The Ancient Theater of Mieza (40°38′38.6″ N 22°07′21.3″ E): DSM (altitudes: from black color 90 m to white color 119 m) without the use of GCPs in the processing of RGB (for example) images; (**b**) the Kasta Mound (40°50′21.5″ N 23°51′44.9″ E): DSM (altitudes: from black color 72 m to white color 107 m) without the use of GCPs in the processing of RGB (for example) images.

**Figure 15:**
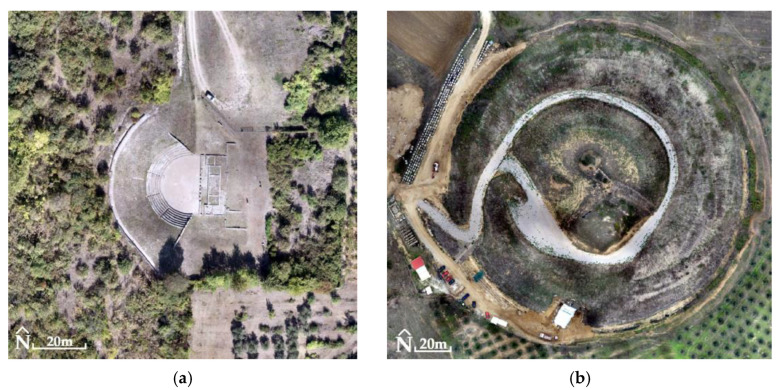
(**a**) The Ancient Theater of Mieza (40°38′38.6″ N 22°07′21.3″ E): orthophotomosaic (true color) without the use of GCPs in the processing of RGB images; (**b**) the Kasta Mound (40°50′21.5″ N 23°51′44.9″ E): orthophotomosaic (true color) without the use of GCPs in the processing of RGB images.

**Figure 16:**
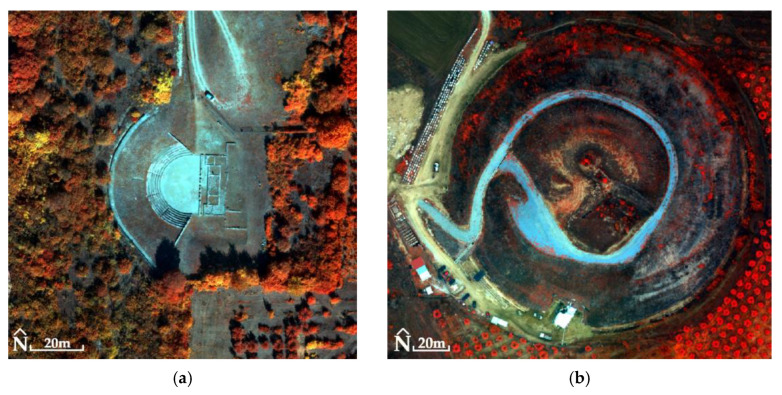
(**a**) The Ancient Theater of Mieza (40°38′38.6″ N 22°07′21.3″ E): orthophotomosaic (NIR, Green, Blue) without the use of GCPs in the processing of MS images; (**b**) the Kasta Mound (40°50′21.5″ N 23°51′44.9″ E): orthophotomosaic (NIR, Green, Blue) without the use of GCPs in the processing of MS images.

For the Ancient Theater of Mieza and the RGB images, RMSE_XYZ_ was 1.4 cm (not using GCPs). The generated products had a spatial resolution of 2.2 cm for DSM (Figure 14) and 1 cm for orthophotomosaic (Figure 15). For MS images, the RMSE_XYZ_ was 0.8 cm (not using GCPs). The generated products had a spatial resolution of 13.5 cm for DSM and 7 cm for orthophotomosaic (Figure 16, Table 3).

For the Kasta Mound and the RGB images, RMSE_XYZ_ was 1.1 cm (not using GCPs). The generated products matched with a spatial resolution of 1.3 cm for DSM (Figure 14) and 0.6 cm for orthophotomosaic (Figure 15). For MS images, the RMSE_XYZ_ was 0.7 cm (not using GCPs). The generated products had a spatial resolution of 14.9 cm for DSM and 7 cm for orthophotomosaic (Figure 16, Table 3).

### 5.2. Process for Checking the Measuring Accuracy of Products

For the Acropolis of Platanias, the RGB images were processed twice, once with the use of GCPs and once without the use of GCPs. For each of the two processing cases, the final products produced were DSM and orthophotomosaic. By extracting the coordinates (x’, y’ and z’) of the CPs from the products, for both processing cases, it was possible to compare them with the coordinates (x, y, z) of the CPs in the field to evaluate the quality of the products (DSM and orthophotomosaic). The mean value, the standard deviation and the analysis of variance were the tools used for this purpose.

The mean value refers to the value of the sum of the differences between the coordinates of the CPs drawn from the products and their corresponding field measurements, divided by the number of CPs. Since the calculation of the mean is not sufficient to draw safe conclusions, the standard deviation was also calculated. The standard deviation is used to determine the range of dispersion of Δx, Δy and Δz from their mean values. Obviously, the values of the standard deviations ought to be as small as possible, and certainly smaller than the corresponding mean values. The analysis of variance (ANOVA) performs hypothesis tests to determine the differences in the mean values of different data sets. Hypothesis H_0_ assumes that all samples come from two different data sets (x-measurement in product and x-measurement in field, y-measurement in product and y-measurement in field, z-measurement in product and z-measurement in field) with the same mean value. The alternative H_A_ hypothesis assumes that at least their mean values are different. When the *p*-value is greater than the constant of 0.05 for a 95% confidence level, then there is no systematic error between the mean values derived from x’ (or y’ or z’) of the products and the actual mean values of these x’ (or y’ or z’, respectively) measured in the field. Thus, any differences between them are considered negligible and are attributed to random errors. When the values of the test statistic F are less than the critical values (F crit), then the standard deviations between x’ (or y’ or z’) and x (or y or z, respectively) do not differ significantly, so that the measurements (field and product) are accompanied only by random errors [8]. Tables with the mean values, standard deviations (Table 4) and the analysis of variance (ANOVA) (Table 5 and Table 6) are presented below (apart from the standard histogram that helped visualize the distribution of the data; we also carried out a number of specific diagnostics such as equality of variances, Skewness and Kurtosis tests; they all pointed to the conclusion that we were dealing with normally distributed data and we therefore proceeded with the ANOVA), and refer to the 3D coordinates of the CPs extracted from the products and compared with the 3D coordinates measured in the field on the corresponding CPs.

### 5.3. Fused Image Production Process and Control of Thematic Information

The MS sensor (RedEdge-MX) does not include a PAN sensor. Below are the satellite image processing procedures, where the satellites are equipped with a PAN sensor that is utilized in image fusion realization, and the RGB orthophotomosaics of the RBG sensor (RX1R II) are transformed into Pseudo-Panchromatic (PPAN) orthophotomosaics [57,58].

The transformation in Photoshop resulted in the production of black and white (B/W) images, where the intensity value of each pixel stems from maintaining the specific brightness percentages of each band (Red, Green and Blue; details of the algorithm used by Photoshop are not known due to copyright restrictions). Apparently, the PPAN images are not spectrally identical to the PAN images of a sensor that is sensitive to the visible area of the spectrum. Until now, techniques for transforming RGB images into B/W images have been developed based on the optimum visual perception of B/W images by the human eye [59,60,61,62] and not on the spectral approach of real PAN images.

**Figure 17:**
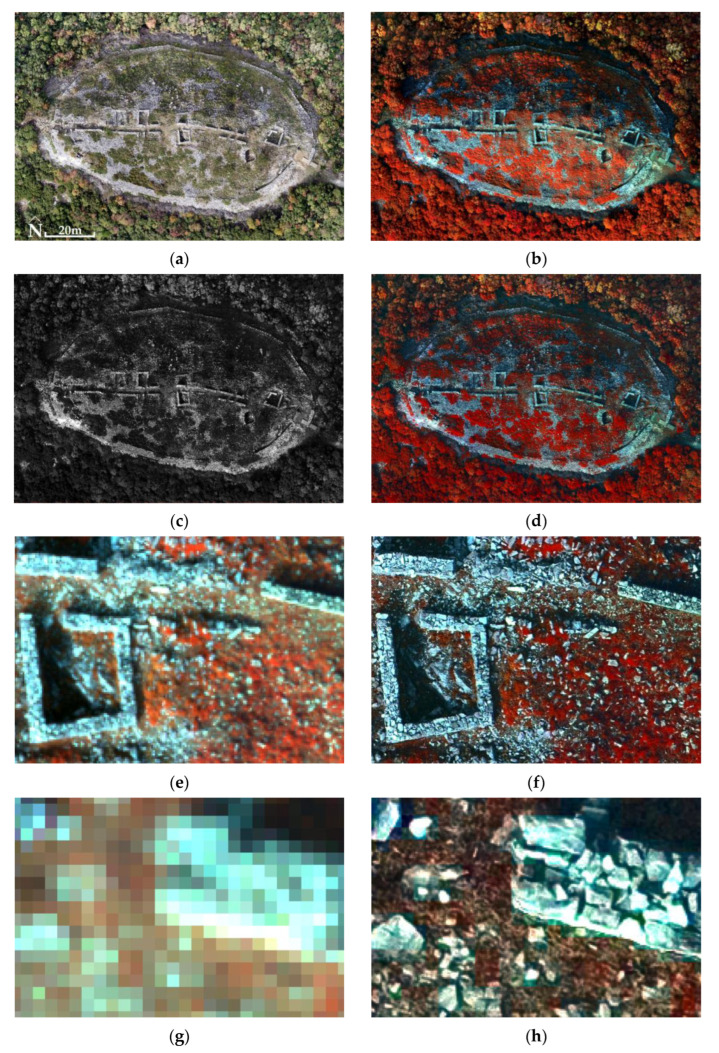
The Acropolis of Platanias (41°11′05.4″ N 24°26′03.2″ E): (**a**) the orthophotomosaic (true color) of the RGB sensor; (**b**) the MS orthophotomosaic (NIR, Green, Blue); (**c**) the PPAN orthophotomosaic; (**d**) the fused image (PCA5, PCA2, PCA1); (**e**,**g**) MS images with spatial resolution 8 cm (in the center of the study area the widths of the walls are between 0.5 m and 0.7 m) and (**f**,**h**) fused images with spatial resolution 1 cm were added at this point to show how important the improvement of the spatial resolution of the MS images is; (**e**,**h**) enlargements at the limit of the pixel size (in the other archaeological sites, corresponding figures are missing, as it is necessary to avoid the unnecessary presentation of archaeological information in high spatial resolution).

**Figure 18:**
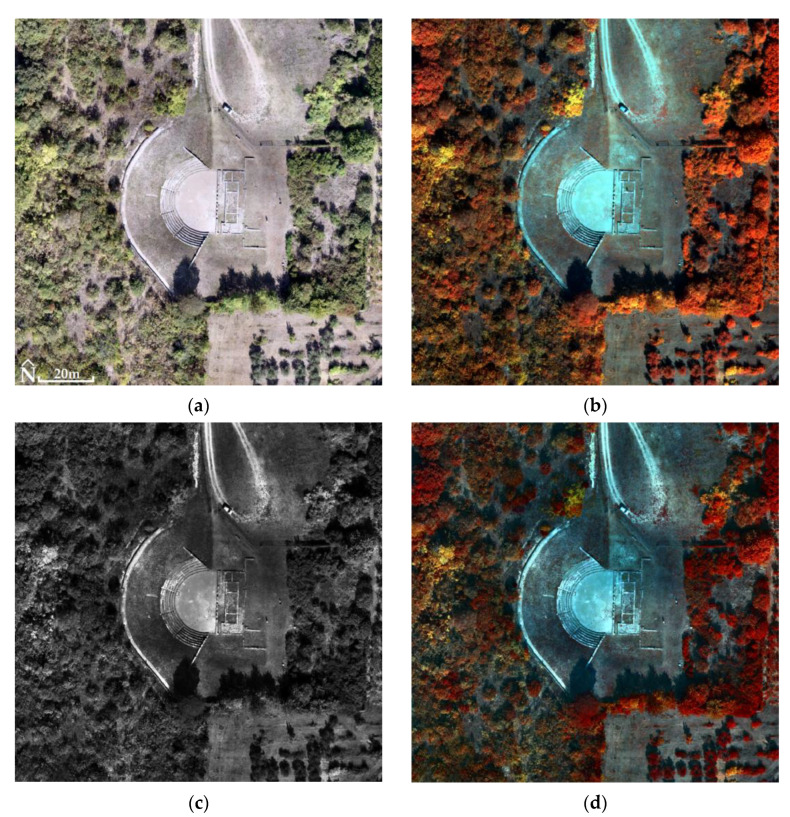
The Ancient Theater of Mieza (40°38′38.6″ N 22°07′21.3″ E): (**a**) the orthophotomosaic (true color) of the RGB sensor; (**b**) the MS orthophotomosaic (NIR, Green, Blue); (**c**) the PPAN orthophotomosaic; (**d**) the fused image (PCA5, PCA2, PCA1).

**Figure 19:**
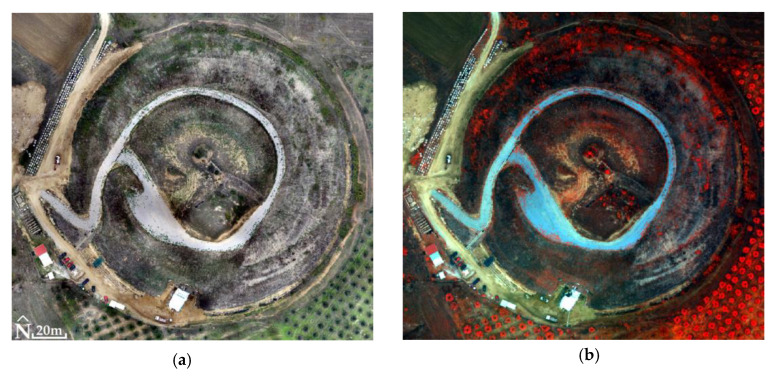

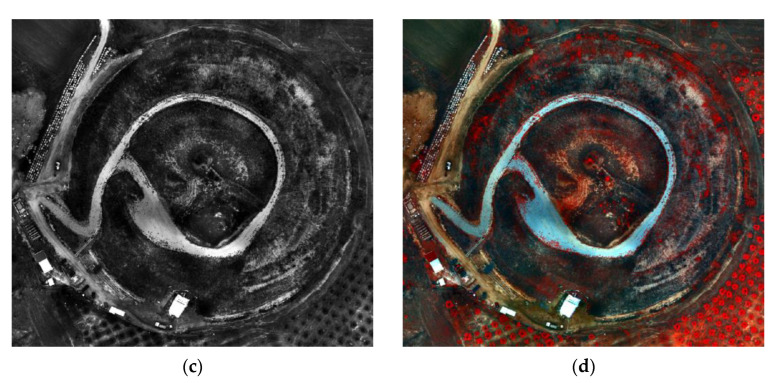
The Kasta Mound (40°50′21.5″ N 23°51′44.9″ E): (**a**) the orthophotomosaic (true color) of the RGB sensor; (**b**) the MS orthophotomosaic (NIR, Green, Blue); (**c**) the PPAN orthophotomosaic; (**d**) the fused image (PCA5, PCA2, PCA1).

Subsequently, the histogram of each PPAN orthophotomosaic was adjusted to the histogram of the corresponding MS orthophotomosaic (Figure 17, Figure 18 and Figure 19). The fused images (Figure 17, Figure 18 and Figure 19) were created using the Principal Component Analysis (PCA) method. In terms of the output produced, any fused image B*h should be as identical as possible to the image Bh that the corresponding sensor would observe with the highest resolution h, if existent. Therefore, the correlation tables (Table 7, Table 8 and Table 9) of the original MS orthophotomosaics with the fused images reveal the retention rate of the original spectral information (which should be >90%, i.e., >0.9) [63,64,65,66,67] (two other techniques, Multiplacative and Brovey Transform, have also been used [66,67,68,69,70], which did not give better results as to the retention of spectral information, and therefore are not analyzed in the paper).

The widespread ERGAS index (Erreur Relative Globale Adimensionnelle de Synthese or Relative Adimensional Global Error in Synthesis), Equation (1), [63] is used to evaluate the quality (quantitative measurement) of the fused image with respect to the MS orthophotomosaic.

(1) $$ERGAS=100\frac{h}{l}\sqrt{\frac{1}{N}\sum_{k=1}^{N}\left[\frac{RMSE(B_{k}{)}^{2}}{(M_{k}{)}^{2}}\right]}$$

where h is the spatial resolution of the high-resolution (fused) images, I is the spatial resolution of the low-resolution (MS) images, N denotes the number of spectral bands and k denotes the index of each band. The RMSE for the k band between the fused and the MS image is shown through RMSE (Bk) (Equation (2)). In the MS image, M_k_ represents the mean of the k-band.

(2) $$RMSE(B)=\sqrt{\frac{\sum_{i=1}^{n}{\left(P_{i}-O_{i}\right)}^{2}}{n}}$$

Values for each spectral band, Pi for MS and Oi for fused images, arise after the selection of random pixels (number of pixels n) at the same coordinates of images.

The limits of the ERGAS index values, which determine the quality of the fused image, are not fixed. They may vary depending on the requirements of each application. For example, when high spectral resolution of images is necessary, then very small index values may be required. In other cases, moderate index values may be acceptable, especially if some factors affect the quality of the fused image (e.g., heavy cloud cover, high levels of atmospheric humidity, etc.). Additionally, the limits of the index are highly dependent on the number and distribution of pixels to be tested (there is no suggested percentage of all pixels of the fused image to be tested), but also on the estimated degree of error acceptance between the two images, which is set solely by the researcher on a case-by-case basis. It follows from the literature that, in general, small index values, close to 0, indicate low relative error between the fused image and MS orthophotomosaic. Therefore, in this case we are dealing with a high-quality fused image. Moderate index values, 0.1 to 1, indicate a moderate relative error. Fused images may be accepted, but there may be small spectral differences between the images (fused image and MS orthophotomosaic). High index values, 1 to 3, indicate high relative error. In this case we are dealing with a low-quality fused image, which differs significantly from the MS orthophotomosaic. All the above limits may, as mentioned above, be modified but in any case, the index values should be less than three in order for a fused image to be characterized in terms of its quality and/or used for classification [63,71,72,73,74,75,76,77,78,79,80,81,82,83,84].

In the Acropolis of Platanias, 31 million of the 120 million pixels of the fused image were checked (using the Model Maker of Erdas Imagine 2015^©^ software to calculate the ERGAS index). The ERGAS index value was 2.8, so there appeared to be a high relative error between the fused image and MS orthophotomosaic. The fused image had a high spectral deviation from the MS orthophotomosaic; therefore, its quality was low.

In the case of the Ancient Theater of Mieza, 54 million of the 169 million pixels of the fused image were examined. The ERGAS index value was 0.5, so there appeared to be a moderate relative error between the fused image and MS orthophotomosaic. The fused image had a moderate spectral deviation from the MS orthophotomosaic, so its quality was good.

Finally, in the case of Kasta Mound, 123 million of the 1 billion pixels of the fused image were examined. The ERGAS index value was 0.2, so there appeared to be a low relative error between the fused image and MS orthophotomosaic. The fused image had a small spectral deviation from the MS orthophotomosaic; therefore, its quality was high.

## 6. Discussion

### 6.1. Measurement Content

If this paper was aimed at the production of an, e.g., orthophotomosaic with the best possible spatial accuracy, then we would want better accuracy in the GCPs than the pixel size of the images (or accuracy of the GCPs at least two or three times better than the ground sampling distance (GSD) of the images). The GSD of RGB images is about 8 mm; this means that the accuracy of the GCPs should be 3–4 mm. On the one hand, this product is not the aim of the paper; on the other hand, unfortunately this accuracy in GCPs cannot be achieved with RTK and PPK technologies (for the correction of location data after they are collected and uploaded), which are used in this paper.

Furthermore, the possibility of an internal block adjustment avoiding external observations––as in the case of direct georeferencing (that is, by processing not using GCPs), and the case of processing using GCPs where external observation GCPs have an accuracy two or three times better than the GSD––may not allow the comparison of the products of both methods (using or not using GCPs).

Therefore, in this paper, the accuracy of the resulting products is checked against the existing accuracies of the GCPs and the centers of the images. The same GNSS is used to measure the GCPs and CPs, and to calculate the coordinates of the images’ centers. These coordinates have approximately the same accuracies (we are in the same area and the measurements are made from the same permanent stations). The question is, with these accuracies, what is the accuracy of the products either with the use of GCPs or without the use of GCPs (direct georeferencing)? With these accuracies, should we use GCPs in the field or can we obtain better products just with the UAS’s PPK system measurements? In the following paragraphs, there is a discussion about the metric content and comparison of the products.

In both cases of processing (using or not using GCPs) of the RGB sensor images, the *p*-values are greater than the constant 0.05 (Table 5 and Table 6), so for a 95% confidence level there appears to be no systematic error between the mean x (or y or z) values of the CPs of the products and the (actual) mean x (or y or z, respectively) values of the CPs measured in the field. Thus, any differences between them are considered negligible and are attributed to random errors. Moreover, in both cases, the values of the test statistic F are below the critical values (F crit), so the standard deviations of x’ (or y’ or z’) and x (or y or z, respectively) do not differ significantly, so that the measurements (field and product) are accompanied only by random errors.

Therefore, the first positive point is that the measurements of CPs (in products and in the field) are not accompanied by systematic errors. Thus, it makes sense to check for the CPs of the means and standard deviations of the differences between the 3D coordinates measured on the products and the 3D coordinates measured in the field.

According to Table 4, the standard deviations of the differences in CPs are smaller than their mean values on all three axes in both processing cases (using or not using GCPs). Therefore, a second positive aspect is that there are small dispersions of Δx, Δy and Δz around their mean values.

A third positive note is that the average values of the CPs’ differences on the horizontal axis are similar and noticeably small, about 1.2 cm, in both processing cases (using or not using GCPs). This implies that the horizontal accuracy of orthophotomosaics is approximately the same and particularly good, whether the processing is performed with or without GCPs. Additionally, the horizontal accuracy is similar to the expected, 1 cm, according to the UAS manufacturer, in the case of RGB sensor image processing without using GCPs [10]. Comparing the above result (1.2 cm) with the values in Table 3, it can be seen that the calculated accuracy values of the CPs are inferior to the software accuracy values in the case of processing without GCPs, and better than the software accuracy values in the case of processing with GCPs. This is understandable since, as already mentioned, the software accuracy values paint a general picture of the accuracy of the products.

A fourth positive note is that in the case of RGB sensor image processing using GCPs, the average value of the CPs’ differences in the vertical axis, 4.5 cm, is that which is theoretically expected (about three times lower than the horizontal accuracy) and very small. However, this is not the case in the case of processing without the use of GCPs. The average value of the CP differences is 7.6 cm, which is below the mean value of the processing using GCPs. In general, this can be described as good, but it does not meet the expected value (3 cm) according to the UAS manufacturer for the case of RGB sensor image processing without using GCPs [10]. In general, the above values, 4.5 cm and 7.6 cm, are below the software accuracy values (Table 3). The large errors on the vertical axis can be reduced (up to half) if a small number of GCPs are used simultaneously in the solution, or if a quality sensor pre-calibration is used from the start, or if more than one GNSS base stations are used to calculate the average of the PPK measurement corrections [4,5,10,17,18,21].

There was no corresponding measurement investigation for the MS sensor. The images of the RGB sensor have a spatial resolution of about 1 cm for a flight height of 60 m and therefore it is possible to identify and mark the GCPs with very good accuracy. Therefore, it is fair to compare the products resulting from the processing of the images with and without the use of GCPs. In the case of the MS sensor, the spatial resolution of the images is about 7 cm for a flight height of 100 m. According to the spatial resolution, it is not possible to identify and mark GCPs with high accuracy, and therefore it would not be fair to compare the products obtained from processing the images with and without the use of GCPs.

### 6.2. Thematic Content

The remark of Figure 17e–h highlights the need to improve the spatial resolution of the MS images of the UAS, which are collected even from a low flight height (e.g., 100 m). In particular, the spatial resolution of MS orthophotomosaics can be improved seven or even eight times in fused images. However, the question of interest when improving the spatial resolution is whether the spectral information of the MS orthophotomosaics is preserved in the fused images.

According to the correlation tables (Table 7, Table 8 and Table 9), the spectral information of the MS orthophotomosaics is transferred to the fused images at a rate of 77% to 91%, with an average of 83% for all correlations of the respective bands for all three archaeological sites. On top of that, the average percentage of the spectral information of the NIR bands transferred from the MS orthophotomosaics to the fused images is 85%. In general, when a percentage below 90% is observed in any correlation of corresponding bands, then the fused image as a whole is not acceptable for classification. On the other hand, the above percentages are objectively not very low and therefore another index should be used that can calculate the spectral difference between the two images. The values of the ERGAS index could be evaluated in combination with the correlation tables to obtain a more reliable conclusion about the classifiability of the fused images.

## 7. Conclusions

With 20 years of academic research experience in the construction (Remote Control-RC Helicopter in 2004, RC Balloon in 2011 and RC hexacopter in 2017 [85]) and use of UAS in photogrammetry and remote sensing applications, a brief, comprehensive view of UAS shall be presented first. The WingtraOne GEN II is an extremely stable and reliable flight system, easy to use and with easily processed raw data (RGB, MS images and PPK system measurements). It covers large areas in a short flight time and is capable of capturing high resolution RGB and MS images.

Orthophotomosaic generation from the RGB sensor images after processing them without using GCPs, with horizontal accuracy similar to the accuracy of classical image processing using GCPs, was applied. Furthermore, the calculated horizontal accuracy (without using GCPs) is in line with the accuracy reported by the UAS manufacturer [10]. This is particularly important, as it can allow corresponding applications to minimize the time spent in the field, since no GCPs are placed and measured. Considering that in challenging terrain, the positioning and measurement of GCPs is not an easy process, the above positive finding is further strengthened.

The vertical accuracy obtained by processing the RGB sensor images without using GCPs is twice lower than the theoretically (according to the calculated horizontal accuracy) expected accuracy or the accuracy obtained by processing the RGB sensor images using GCPs (i.e., it seems that the classical image processing procedure using GCPs gives a better result). This vertical accuracy does not seem to affect the horizontal accuracy of the orthophotomosaic of the RGB sensor, but only accompanies the generated DSM of the RGB sensor. In corresponding image processing studies without the use of GCPs [4,11,12,13,14,15,16,17,18], similar or larger differences in vertical accuracy were calculated on the one hand, while on the other hand a noticeable improvement in vertical accuracy, at least up to 50%, is observed in the different regions under study (obviously, in these studies more than one region is studied), and thus the very large difference in vertical accuracy calculated in one region can be considered accidental.

The horizontal and vertical accuracies calculated in this study during the processing of the RGB sensor images without the use of GCPs cannot substantiate the actual accuracy that can be achieved, since on the one hand the research was carried out in only one area (the Acropolis of Platanias), and on the other hand a quality sensor pre-calibration was not employed. They are the first in a series of identical observations already planned in the short term at more archaeological sites, which will allow safe conclusions to be drawn. Furthermore, more than one GNSS base station will be used in the new applications to calculate the average of the corrections of the initial PPK measurements, as well as a quality sensor pre-calibration.

For the Ancient Theater of Mieza and the Kasta Mound, the correlation tables (Table 8 and Table 9) show that the spectral information transferred from the MS orthophotomosaics to the corresponding fused images (correlation test of corresponding bands) is slightly below the 90% threshold (specifically, the average for all correlations of corresponding bands for both archaeological sites is 83%). In addition, the ERGAS index values are 0.5 for the Ancient Theater of Mieza and 0.2 for the Kasta Mound, which means that the fused images are of good and high quality, respectively, as the spectral deviations (between fused images and MS orthophotomosaics) are at a moderate and low level. Combining the above, the two fused images can be used for classification.

Concerning the Acropolis of Platanias, the correlation table (Table 7) shows that the spectral information carried is slightly below the 90% threshold (the average for all correlations of corresponding bands is 82%). However, the ERGAS index value of 2.8 (just below the safety threshold) reveals that the fused image is of low quality and therefore cannot be used for classification.

The improvement of the spatial resolution of the MS orthophotomosaics by producing fused images suitable for classification at two of the three archaeological sites shows that image fusion can be achieved by utilizing the images of the two sensors, the Sony RX1R II (RGB sensor) and MicaSense RedEdge-MX (MS sensor). This remains to be confirmed again in the new observations already planned in the short term at other archaeological sites.

## Figures and Tables

**Table 1:** Technical characteristics of the RGB and MS sensor [10,46] of the UAS.

Sensor	Technical Specifications
Sony RX1R II	RGB sensor
	Full frame sensor
	Focal Length 35 mm
	42.4 Mpixel (resolution 7952 × 5304)
	Weight: 590 g
	Ground Sample Distance: 1.6 cm/pixel at 120 m
	Field of View (FOV): 56.2° Horizontal FOV; 39.2° Vertical FOV
MicaSense RedEdge-MX	Multispectral sensor
	Focal length 5.5 mm
	1.2 Mpixel (resolution 1280 × 960)
	Weight: 231.9 g (includes DLS 2 and cables)
	5 spectral cameras: Blue (465–485 nm); Green (550–570 nm); Red (662–673 nm); Red Edge (712–722 nm); Near Infrared (NIR) (820–860 nm)
	Ground Sample Distance: 8.2 cm/pixel at 120 m
	Field of View (FOV): 47.2° Horizontal FOV; 36.2° Vertical FOV

**Table 2:** Coordinates of GCPs and CPs in the Greek Geodetic Reference System 87 (GGRS87).

GCPs				CPs			
	X	Y	Z		X	Y	Z
	Meters				Meters		
1	536,326.21	4,559,063.30	670.83	2	536,325.01	4,559,065.66	671.19
5	536,313.18	4,559,073.79	667.88	3	536,309.19	4,559,059.89	667.74
9	536,306.36	4,559,084.46	667.63	6	536,313.04	4,559,078.68	668.57
12	536,289.30	4,559,078.67	669.74	7	536,298.45	4,559,067.59	668.69
13	536,291.56	4,559,084.80	668.12	10	536,301.55	4,559,086.66	667.07
15	536,278.47	4,559,083.60	667.48	11	536,290.01	4,559,076.18	669.40
20	536,267.93	4,559,078.29	666.24	14	536,287.09	4,559,084.78	667.92
21	536,250.96	4,559,081.51	662.60	19	536,267.14	4,559,076.95	665.72
23	536,256.40	4,559,090.84	660.66	23	536,256.40	4,559,090.84	660.66
26	536,239.48	4,559,078.17	660.71	25	536,238.34	4,559,080.25	661.10
27	536,227.14	4,559,077.55	656.86	28	536,223.19	4,559,078.39	656.38
30	536,230.70	4,559,066.69	658.42	29	536,228.90	4,559,069.64	658.43
31	536,251.49	4,559,070.49	662.35	33	536,258.67	4,559,070.06	664.19
35	536,267.90	4,559,087.63	664.02	36	536,271.48	4,559,085.62	665.47
37	536,273.71	4,559,067.20	665.89	38	536,278.18	4,559,071.03	667.08
39	536,295.05	4,559,058.31	666.19	42	536,304.92	4,559,051.79	667.17
41	536,299.44	4,559,049.99	665.76	46	536,239.97	4,559,093.23	655.72
43	536,259.73	4,559,061.76	659.86	47	536,278.86	4,559,096.42	661.20
44	536,289.36	4,559,099.56	660.02	48	536,281.73	4,559,057.27	662.67
45	536,268.66	4,559,103.77	655.56	49	536,247.49	4,559,060.65	659.20

**Table 3:** Analysis results in Agisoft Metashape Professional^©^ and the spatial resolutions of the products.

Scope	Sensor	Use of	RMSE_XY_	RMSE_Z_	RMSE_XYZ_	DSM	Ortho
			cm				
Acropolis of Platanias	RGB	GCPs	1.7	1.7	2.4	2.1	1
	RGB	PPK	0.8	0.9	1.2	2.1	1
	MS	PPK	0.6	0.9	1.1	16.7	8
Theater of Mieza	RGB	PPK	1.0	0.9	1.4	2.2	1
	MS	PPK	0.4	0.7	0.8	13.5	7
Kasta Mound	RGB	PPK	0.7	0.8	1.1	1.3	0.6
	MS	PPK	0.4	0.6	0.7	14.9	7

**Table 4:** Mean values and standard deviations of CPs for the two processing cases.

Processing Cases	CPs (x’, y’, z’ Values in Products—x, y, z Field Measurements)					
	Δx = │x’ − x│		Δy = │y’ − y│		Δz = │z’ − z│	
	Average Value	Standard Deviation	Average Value	Standard Deviation	Average Value	Standard Deviation
	cm					
Without the use of GCPs	1.1	0.9	1.2	1.0	7.6	5.0
With the use of GCPs	1.3	0.8	1.2	1.0	4.5	3.5

**Table 5:** ANOVA. Comparison of x and x’, y and y’ and z and z’ of CPs (without using GCPs).

Source of Variation		Sum of Squares	Degrees of Freedom	Mean Square	F	*p*-Value	F Crit
x and x’	Between Groups	0.000255025	1	0.000255025	2.93168 × 10^−7^	0.999570818	4.09817173
	Within Groups	33055.95674	38	869.8935984			
	Total	33055.95700	39				
y and y’	Between Groups	0.000855625	1	0.000855625	5.52466 × 10^−6^	0.998136903	4.098171731
	Within Groups	5885.208865	38	154.8739175			
	Total	5885.209721	39				
z and z’	Between Groups	0.0390625	1	0.0390625	0.00188425	0.965603638	4.098171731
	Within Groups	787.7802099	38	20.73105816			
	Total	787.8192724	39				

**Table 6:** ANOVA. Comparison of x and x’, y and y’ and z and z’ of CPs (using GCPs).

Source of Variation		Sum of Squares	Degrees of Freedom	Mean Square	F	*p*-Value	F Crit
x and x’	Between Groups	2.24994 × 10^−7^	1	2.24994 × 10^−7^	2.58607 × 10^−10^	0.999987253	4.098171731
	Within Groups	33060.88924	38	870.023401			
	Total	33060.88924	39				
y and y’	Between Groups	0.000455625	1	0.000455625	2.94232 × 10^−6^	0.998640348	4.098171731
	Within Groups	5884.387443	38	154.8523011			
	Total	5884.387899	39				
z and z’	Between Groups	0.008850625	1	0.008850625	0.000425089	0.983658519	4.098171731
	Within Groups	791.1848462	38	20.82065385			
	Total	791.1936968	39				

**Table 7:** Correlation table, Acropolis of Platanias.

		MS Orthophotomosaic					Fused Image (FI)				
		Bands									
		1	2	3	4	5	1	2	3	4	5
MS	1	1	0.934	0.931	0.631	0.290	*0.847*	0.802	0.814	0.615	0.314
	2	0.934	1	0.932	0.818	0.515	0.736	*0.776*	0.744	0.732	0.506
	3	0.931	0.932	1	0.743	0.383	0.753	0.752	*0.825*	0.682	0.370
	4	0.631	0.818	0.743	1	0.846	0.361	0.465	0.440	*0.786*	0.756
	5	0.290	0.515	0.383	0.846	1	0.001	0.098	0.039	0.548	*0.864*
FI	1	*0.847*	0.736	0.753	0.361	0.001	1	0.953	0.950	0.654	0.262
	2	0.802	*0.776*	0.752	0.465	0.098	0.953	1	0.947	0.782	0.380
	3	0.814	0.744	*0.825*	0.440	0.039	0.950	0.947	1	0.728	0.286
	4	0.615	0.732	0.682	*0.786*	0.548	0.654	0.782	0.728	1	0.767
	5	0.314	0.506	0.370	0.756	*0.864*	0.262	0.380	0.286	0.767	1

**Table 8:** Correlation table, Ancient Theater of Mieza.

		MS Orthophotomosaic					Fused Image (FI)				
		Bands									
		1	2	3	4	5	1	2	3	4	5
MS	1	1	0.948	0.977	0.792	0.535	*0.908*	0.835	0.895	0.678	0.410
	2	0.948	1	0.943	0.929	0.728	0.837	*0.843*	0.843	0.766	0.556
	3	0.977	0.943	1	0.815	0.555	0.879	0.819	*0.907*	0.689	0.418
	4	0.792	0.929	0.815	1	0.901	0.652	0.722	0.687	*0.774*	0.665
	5	0.535	0.728	0.555	0.901	1	0.384	0.497	0.422	0.638	*0.829*
FI	1	*0.908*	0.837	0.879	0.652	0.384	1	0.960	0.980	0.820	0.568
	2	0.835	*0.843*	0.819	0.722	0.497	0.960	1	0.956	0.935	0.734
	3	0.895	0.843	*0.907*	0.687	0.422	0.980	0.956	1	0.845	0.594
	4	0.678	0.766	0.689	*0.774*	0.638	0.820	0.935	0.845	1	0.896
	5	1	0.948	0.977	0.792	*0.535*	0.908	0.835	0.895	0.678	0.410

**Table 9:** Correlation table, Kasta Mound.

		MS Orthophotomosaic					Fused Image (FI)				
		Bands									
		1	2	3	4	5	1	2	3	4	5
MS	1	1	0.950	0.887	0.812	0.441	*0.822*	0.814	0.789	0.736	0.407
	2	0.950	1	0.955	0.929	0.587	0.707	*0.781*	0.772	0.760	0.489
	3	0.887	0.955	1	0.920	0.515	0.640	0.735	*0.793*	0.737	0.399
	4	0.812	0.929	0.920	1	0.754	0.536	0.664	0.684	*0.760*	0.615
	5	0.441	0.587	0.515	0.754	1	0.194	0.322	0.296	0.491	*0.859*
FI	1	*0.822*	0.707	0.640	0.536	0.194	1	0.958	0.911	0.852	0.448
	2	0.814	*0.781*	0.735	0.664	0.322	0.958	1	0.967	0.942	0.552
	3	0.789	0.772	*0.793*	0.684	0.296	0.911	0.967	1	0.935	0.501
	4	0.736	0.760	0.737	*0.760*	0.491	0.852	0.942	0.935	1	0.706
	5	0.407	0.489	0.399	0.615	*0.859*	0.448	0.552	0.501	0.706	1

## Data Availability

The figures in this paper have a print resolution similar to or inferior to the images, e.g., of Google Maps or Google Earth. No original images or raw data will be made available on the locations, as they concern archaeological sites.
